# Dynamic of Cerebrospinal Fluid and Acute-Phase Proteins After Subarachnoid Hemorrhage: The Lumbar Compartment as Blind Spot?

**DOI:** 10.1007/s12028-025-02411-0

**Published:** 2025-11-24

**Authors:** Anton Früh, Ran Xu, Laura Hallek, Nicolas Musigk, Peter Truckenmueller, Clara F. Weber, Kai Seeger, Eleni Balla, Peter Vajkoczy, Stefan Wolf

**Affiliations:** 1 Department of Neurosurgery, Charité Universitätsmedizin Berlin, Corporate Member of Freie Universität Berlin, Humboldt-Universität Zu Berlin and Berlin Institute of Health, Berlin, Germany; 2 Department of Cardiology, Angiology, and Intensive Care Medicine, Deutsches Herzzentrum Der Charité, Berlin, Germany

**Keywords:** Subarachnoid hemorrhage, Cerebrospinal fluid, Compartment, Lumbar drainage

## Abstract

**Background:**

The rupture of an intracranial aneurysm, resulting in aneurysmal subarachnoid hemorrhage (aSAH), constitutes a devastating neurological event marked by an abrupt clinical onset and high morbidity and mortality rates. Controlled lumbar drainage following aSAH has been associated with improved neurological outcomes; however, the underlying mechanisms remain poorly understood. This study aims to characterize the lumbar and cranial cerebrospinal fluid (CSF) compartments and contrast these with serum laboratory test findings, providing a foundational framework for future investigations into the mechanisms by which lumbar drainage facilitates neurological recovery.

**Methods:**

We retrospectively included patients with aSAH for whom CSF specimens were available. Baseline demographic data, serum laboratory test results, and CSF findings were collected following the hemorrhagic event. A CSF infection was defined as a positive microbiological culture result.

**Results:**

We included 454 patients, with a total of 7,176 serum samples and 1,911 CSF samples. All patients showed an initial increase in acute-phase protein levels, with a decline afterward, and these dynamics revealed a significant effect of lumbar drainage. Lower C-reactive protein (CRP) levels were observed when using a lumbar drain (*p* < 0.001). Furthermore, CRP levels peaked earlier and consecutively declined earlier with the use of lumbar drains (*p* < 0.001). Similarly, leukocyte levels were higher in patients without lumbar drainage (*p* = 0.002). The majority of blood cells were detected in the lumbar CSF compartment. Notably, the differences between the lumbar and ventricular CSF compartments persisted for up to five days post hemorrhage.

**Conclusions:**

Ventricular and lumbar CSF compartments exhibit significant differences within the first days following aSAH. These findings suggest that blood cell accumulation in the lumbar compartment may contribute to post-aSAH inflammatory processes. Accordingly, the source of CSF samples should be carefully considered to ensure accurate interpretation and to guide therapeutic decision-making in the management of aSAH.

## Introduction

Rupture of an intracranial aneurysm resulting in aneurysmal subarachnoid hemorrhage (aSAH) constitutes a catastrophic neurological event with an abrupt onset of clinical manifestations and elevated rates of morbidity and mortality [1–4]. Epidemiological data suggest an incidence of approximately nine cases per 100,000 person-years. Available evidence indicates that women are disproportionately affected compared to men [2, 5].

Immunological processes play a significant role in the pathophysiological mechanisms of aSAH [6, 7]. In the management of this severe disorder, the prospective multicenter Earlydrain trial [8] demonstrated that prophylactic controlled lumbar drainage at a rate of 5 mL/hr following aneurysm closure resulted in improved neurological outcomes. The underlying mechanisms for this effect remain unclear. Moreover, a subanalysis of the Earlydrain data indicated that there may even be volume-dependent benefits associated with lumbar drainage [9]. A principal characteristic of aSAH is the presence of blood in the subarachnoid space, which is part of the cerebrospinal fluid (CSF) system—a closed fluid system composed of both ventricular and spinal compartments. We hypothesize that additional lumbar drainage may facilitate the clearance of blood from areas such as the basal cisterns and lumbar compartment, which are not accessible via an external ventricular drain (EVD). This residual blood could exert proinflammatory effects, suggesting that supplementary lumbar washing might attenuate the immune response following aSAH.

In this study, we aimed to investigate the cell count of both the lumbar and ventricular CSF compartments following aSAH and to examine whether additional prophylactic lumbar drainage exerts influence on these parameters.

## Methods

### Study Design and Patients

This is a single-center study. We retrospectively included patients from 2011 to 2023 with aSAH treated at our tertiary care center covering an area of approximately 3.5 million inhabitants. Ethical approval was obtained from the local ethics committee (number: EA1/291/14), and all procedures were conducted in accordance with the principles of the Declaration of Helsinki. Patient management followed previously published protocols [8] and internationally recognized guidelines [10, 11]. The analysis adhered to the Strengthening in the Reporting of Observational studies in Epidemiology (STROBE) criteria.

In all patients, indication for placement of an external ventricular drainage was determined at the discretion of the physician in charge. An additional lumbar drain was inserted per local treatment standard of care after securing the aneurysm in the majority of patients, according to the Earlydrain protocol. After 2018, part of the team was aware of preliminary results from the Earlydrain trial. Reasons not to insert a lumbar drain were participation of the patient in a prospective trial actively prohibiting the use of lumbar drains, patients in good clinical grade with modified Fisher I and II hemorrhages, or contraindications such as obstructed basal cisterns or signs of herniation in the initial or a follow-up computed tomography (CT) scan. Patients for whom no CSF data were available, those under 18 years of age, and those who died within the first two days after the bleeding event were excluded from the analysis. Patients who initiated lumbar drainage within the first three days and had at least 480 mL of CSF drained were assigned to the lumbar drainage group. This classification is in accordance with the as-treated group definition in the Earlydrain trial [8]. For this retrospective data analysis, no additional CSF or blood samples were collected. The indication for sampling was determined by the treating physician, with possible reasons including the management of intracranial pressure, suspicion of infection, or other clinically relevant considerations. Per local standard operating procedure, after placement of a CSF drain, a baseline analysis of the parameters was suggested. A subset of these patients participated in the Earlydrain trial, in which participants received either standard management or standard management plus a lumbar drain.

### Drainage Management

All drains remained closed as standard protocol and were only opened actively for CSF drainage according to the study by Olson et al. [12]. Indication for drainage of an EVD was an intracranial pressure rise above 20 mm Hg. Indication for a lumbar drain was CSF clearance according to the Earlydrain protocol, with 5 mm/hr as the start and an increase of this rate if suggested by hydrocephalic appearance on follow-up CT scans or repeated intracranial pressure elevations triggering EVD use. The difference between pressure levels from EVD and lumbar drain was continuously determined, with a difference of less than 5 mm Hg indicating a patent CSF pathway and thus the safety of lumbar drainage [13]. In case of a diagnosed infection, drains were routinely removed and replaced if necessary. In Earlydrain, the development of an infection was linked to the presence of an EVD, not to the use of an additional lumbar drain. Therefore, in cases in which both an EVD and a lumbar drain were present, the EVD was removed first after a few days of established patency of CSF pathways.

### Data Collection

Baseline demographic information and serum laboratory and CSF results after the bleeding event were collected, along with descriptive radiological imaging. Physicians involved were not aware of the presence of a lumbar drain. Infections were defined conservatively by the detection of a microorganism in the CSF culture, blood culture, and/or culture of tracheobronchial secretions within the first 14 days after the bleeding event.

### Statistical Analysis

Categorical variables were presented as counts and percentages and analyzed using the *χ*^2^ test. Continuous variables were reported either as means with standard deviations or as medians with interquartile ranges (IQRs) and compared using either the *t*-test or the Mann–Whitney *U*-test, depending on the distribution. Statistical significance was defined as two-sided *p* values < 0.05. All statistical analyses were performed using SPSS (version 25.0, IBM Corp.), R (version 4.3.1, R Foundation for Statistical Computing, Vienna, Austria), RStudio (version 2023.06.0), and GraphPad Prism (version 10, GraphPad Software, Boston, MA). The following packages were used: readxl for importing Excel data, ggplot2 for data visualization, dplyr for data manipulation, and lme4 for linear mixed model (LMM) analysis. Normality testing was conducted using the Shapiro–Wilk test, group comparisons were performed using either an independent *t*-test or a Wilcoxon rank-sum test depending on normality assumptions, and time-dependent analyses were performed using LMMs.

## Results

### Baseline Characteristics

The final study population consisted of 454 patients with a median age of 55 (IQR 46–64) years. The majority of the study participants were female (61%); 100 (22.0%) of the study participants received an additional controlled lumbar drainage. Table 1 provides the baseline characteristics of the study population, stratified based on whether a controlled lumbar drainage was performed or not.

**Table 1:** Baseline characteristics of patients with aneurysmal subarachnoid hemorrhage, stratified according to LD

	Study population, *N* = 454	LD, *n* = 100	no LD, *n* = 354	*p* value
Age, median (IQR), y	55 (47–64)	54 (46–61)	55 (47–64)	0.33
*Sex, n (%)*				
Female	277 (61)	61 (61)	216 (61)	> 0.99
Male	177 (39)	39 (39)	138 (39)	
*Location of aneurysm, n (%)*				
Anterior inferior cerebellar artery	1 (0.2)	0 (0)	1 (0.3)	> 0.99
Anterior cerebral artery	16 (3.5)	2 (2.0)	14 (4.0)	
Anterior com artery	144 (32)	29 (29)	115 (32)	
Basilar artery	231(6.8)	11 (11)	20 (5.6)	
Carotic artery	41 (9.0)	8 (8.0)	33 (9.3)	
Middle cerebral artery	111 (24)	21 (21)	90 (25)	
Multiple aneurysms	18 (4.0)	3 (3.0)	15 (4.2)	
Pericallosal artery	2 (0.4)	0 (0)	2 (0.6)	
Posterior inferior cereballar artery	6 (1.3)	3 (3.0)	3 (0.8)	
Posterior cerebral artery	2 (0.4)	0 (0.0)	2 (0.6)	
Posterior com artery	70 (15)	19 (19)	51 (14)	
Superior cerebellar artery	2 (0.4)	1 (1.0)	1 (0.3)	
Vertebral artery	10 (2.2)	3 (3.0)	7 (2.0)	
*Hunt & Hess score, n (%)*				
1	79 (17)	11 (11)	68 (19)	0.25
2	104 (23)	29 (29)	75 (21)	
3	79 (17)	19 (19)	60 (17)	
4	77 (17)	17 (17)	60 (17)	
5	115 (25)	24 (24)	91 (26)	
*Modified Fisher scale, n (%)*				
I	23 (5.1)	1 (1.0)	22 (6.2)	0.13
II	21 (4.6)	5 (5.0)	16 (4.5)	
III	147 (32)	37 (37)	110 (31)	
IV	263 (58)	57 (57)	206 (58)	
*Treatment, n (%)*				
Clipping	219 (48)	45 (45)	174 (49)	**0.027**
Endovascular	196 (43)	62 (62)	134 (38)	
None	39 (8.6)	3 (3.0)	36 (10)	

IQR, interquartile range; LD, lumbar drainage

### Longitudinal Profile of Laboratory Serum Parameters After aSAH

**Fig. 1:**
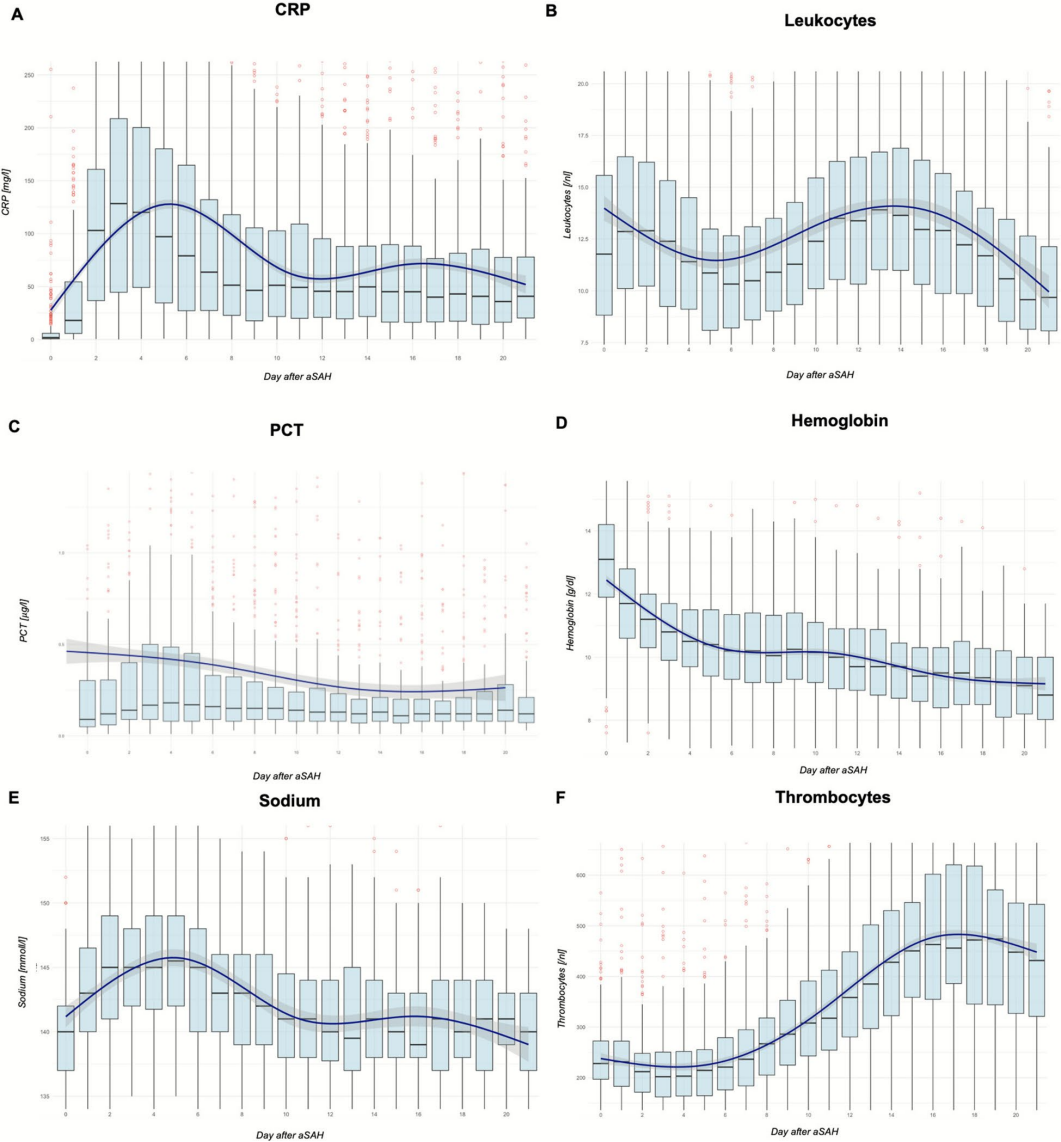
Longitudinal laboratory profile after aneurysmal subarachnoid hemorrhage (aSAH) (7,176 samples from 454 patients). **a**, C-reactive protein (CRP). **b**, Leucocytes. **c**, Procalcitonin (PCT). **d**, Hemoglobin. **e**, Sodium. **f**, Thrombocytes. Data are presented as box plots for the days after aSAH, with outliers highlighted in red. Boxplots show the median values, denoted with a central line, and the boxes indicate the IQR. The mean trend is shown using a Locally Estimated Scatterplot Smoothing curve, with shaded areas indicating the confidence interval

Overall, 7,176 serum samples of 454 patients were included. Figure 1 illustrates the longitudinal course of serum laboratory values for the whole study population. An increase in acute-phase protein levels, including C-reactive protein (CRP) and leukocytes, was observed, peaking during the first and second weeks following the bleeding event. Procalcitonin (PCT) levels remained stable throughout this period. Sodium levels exhibited a peak during the first week of therapy, whereas hemoglobin levels progressively decreased over the initial two weeks. Additionally, patients developed thrombocytosis during the second week post event.

### Longitudinal Profile of CSF Parameters After aSAH

**Fig. 2:**
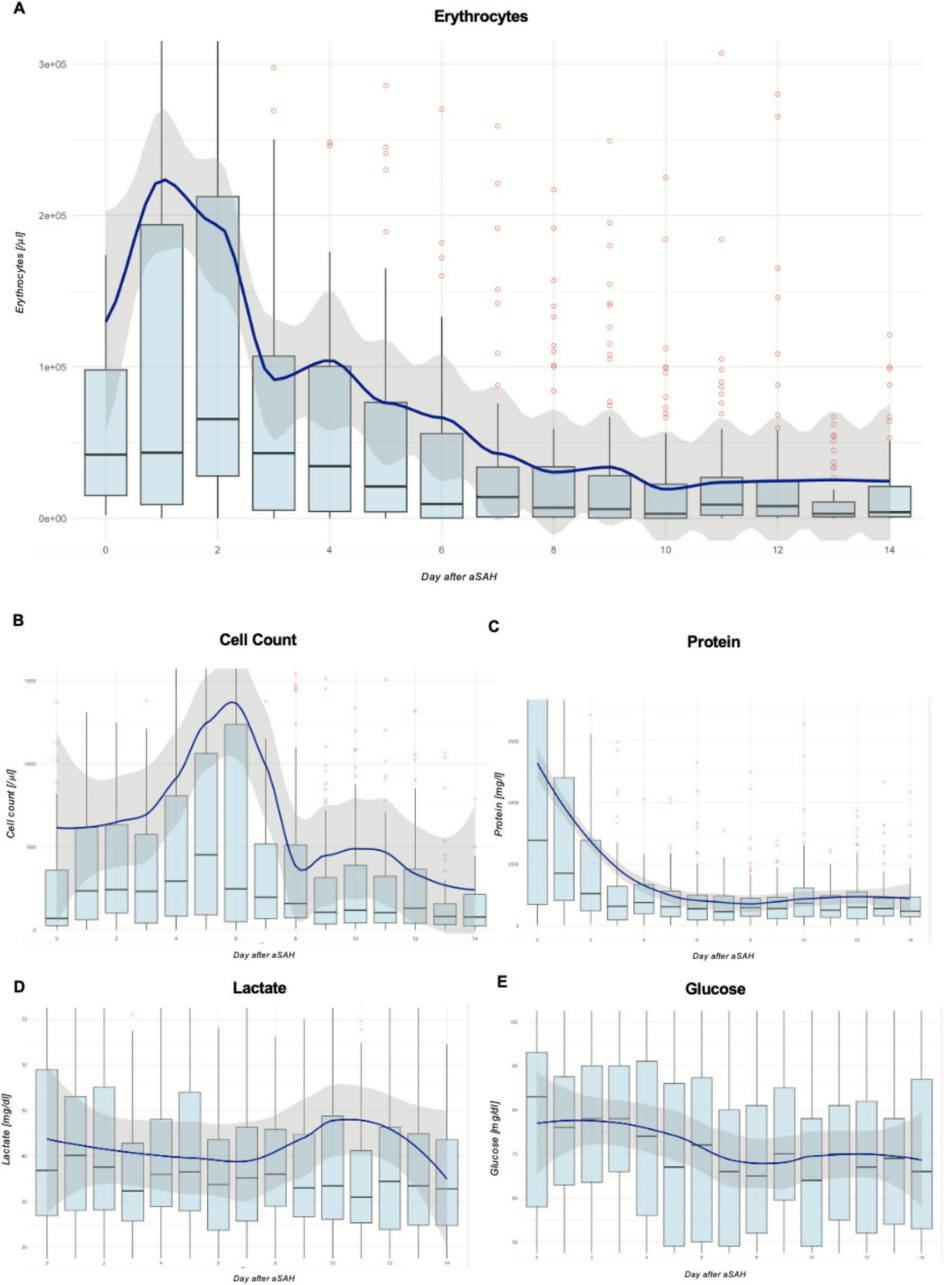
Longitudinal profile of cerebrospinal fluid parameters after aneurysmal subarachnoid hemorrhage (aSAH) (1,911 samples). **a**, Erythrocytes. **b**, Total cell count. **c**, Protein. **d**, Lactate. **e**, Glucose. Data are presented as boxplots for the days after aSAH, with outliers highlighted in red. For a description of boxplots and Locally Estimated Scatterplot Smoothing curves, see the Fig. 1 caption

Overall, 1,911 CSF specimens from 357 patients were analyzed. Of these, 794 samples were collected from the ventricular compartment, and 1,117 samples were obtained from the lumbar CSF compartment. Simultaneous collection of specimens from both the lumbar and ventricular compartments was performed for 415 samples. Figure 2 provides the longitudinal profile of all CSF parameters of the study population. The data show a decrease of erythrocyte levels within the first week after the bleeding event.

### Ventricular and Lumbar CSF Compartments Differ After aSAH

**Fig. 3:**
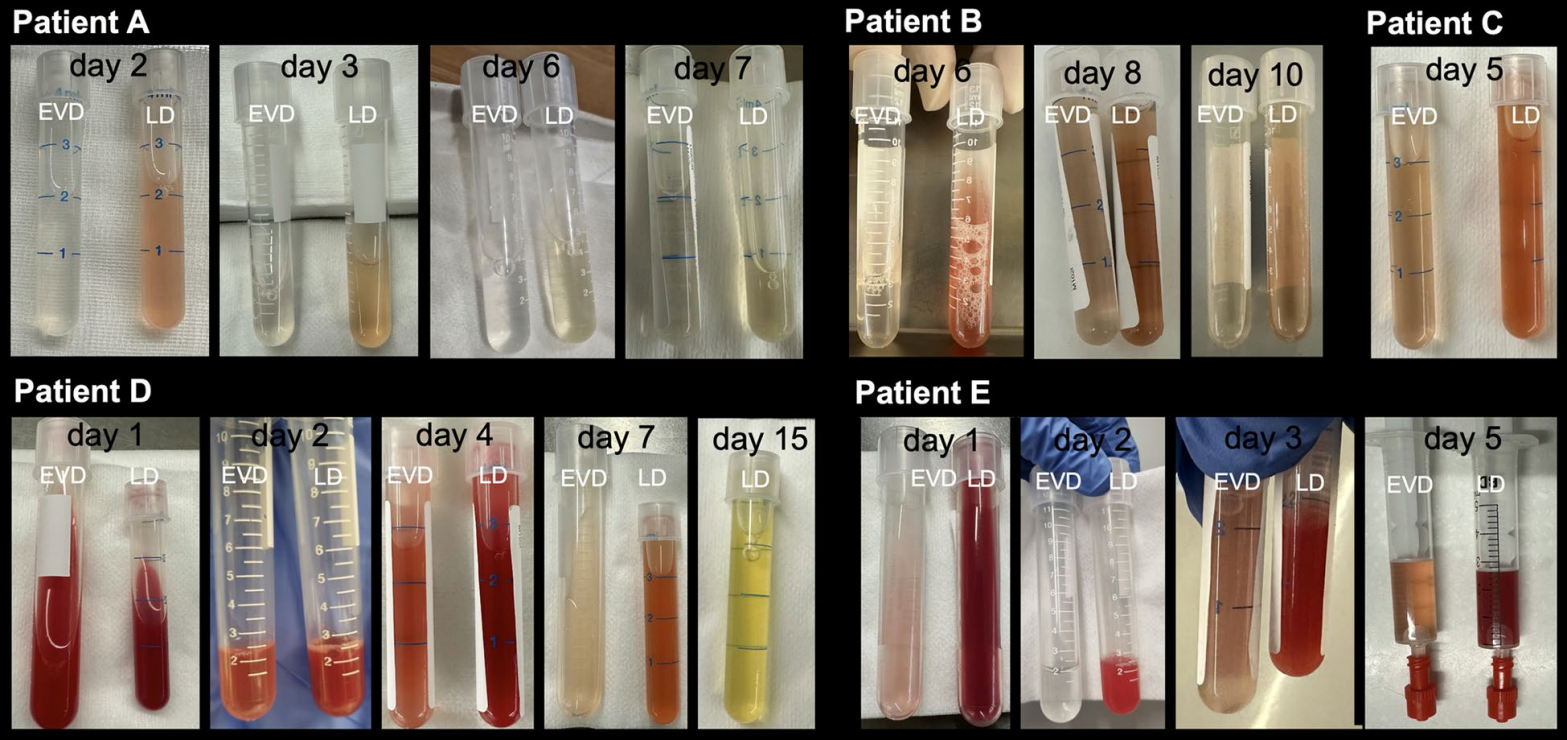
Cerebrospinal fluid specimens. Day = day after bleeding event

**Fig. 4:**
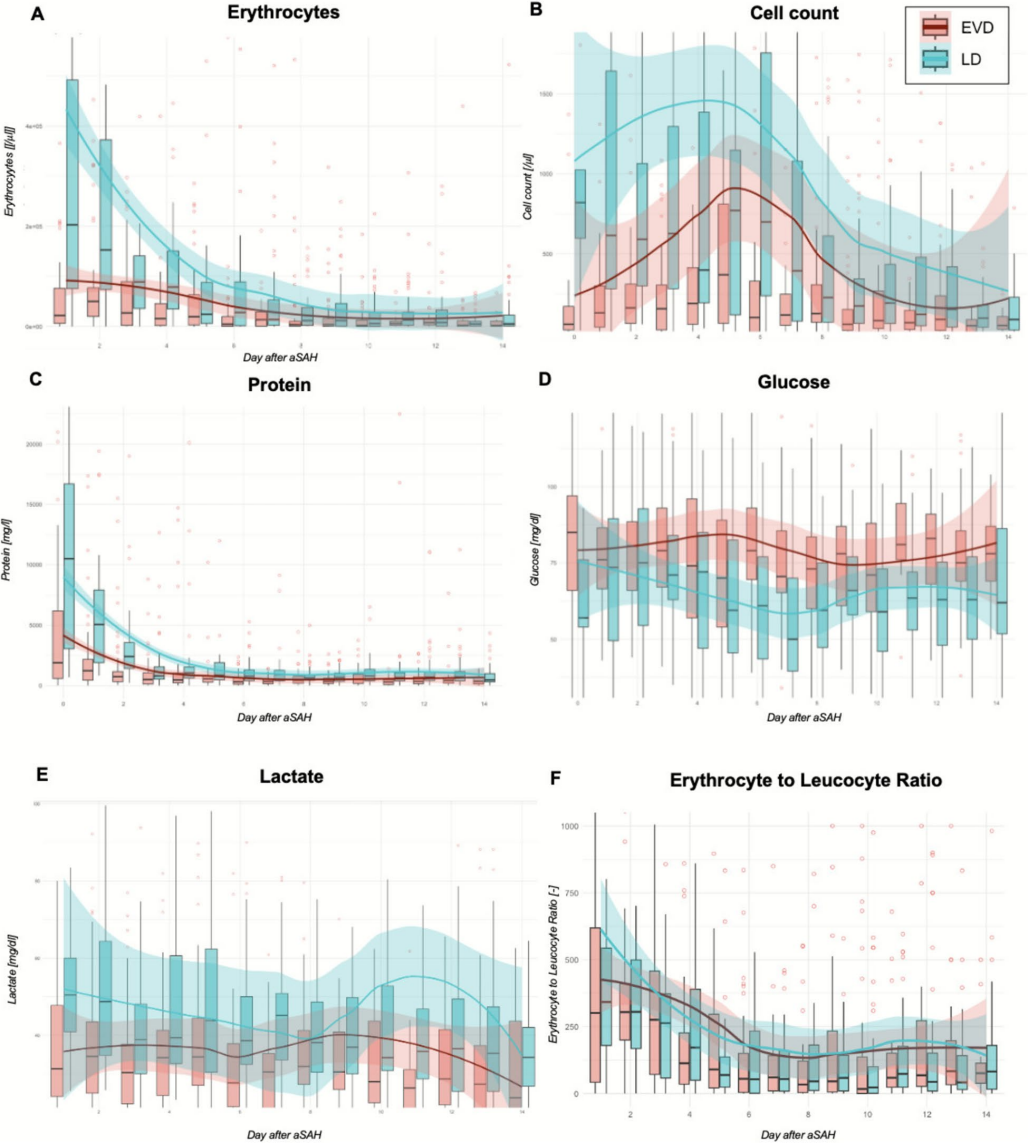
Profile of cerebrospinal fluid parameters after aneurysmal subarachnoid hemorrhage (aSAH) stratified by lumbar and ventricular compartment. **a**, Erythrocytes. **b**, Total cell count. **c**, Protein. **d**, Glucose. **e**, Lactate. **f**, Erythrocyte to leucocyte ratio. Data are presented as boxplots for the days after aSAH, with outliers highlighted in red. The median trend is shown using a Locally Estimated Scatterplot Smoothing curve, with shaded areas indicating the confidence interval. EVD external ventricular drain, LD lumbar drain

Images of CSF samples from different compartments are shown in Fig. 3. Macroscopically, in patients in whom CSF was simultaneously collected from both the ventricular and lumbar compartments, the lumbar compartment appears significantly darker, indicative of a higher concentration of blood cells. The CSF parameters after aSAH, stratified by lumbar and ventricular compartments, are presented in Fig. 4. These data demonstrate that the lumbar compartment shows a higher cell count, with erythrocyte and protein levels requiring approximately five days to equilibrate between the compartments.

### Influence of Additional Lumbar Drainage on Acute-Phase Proteins

**Fig. 5:**
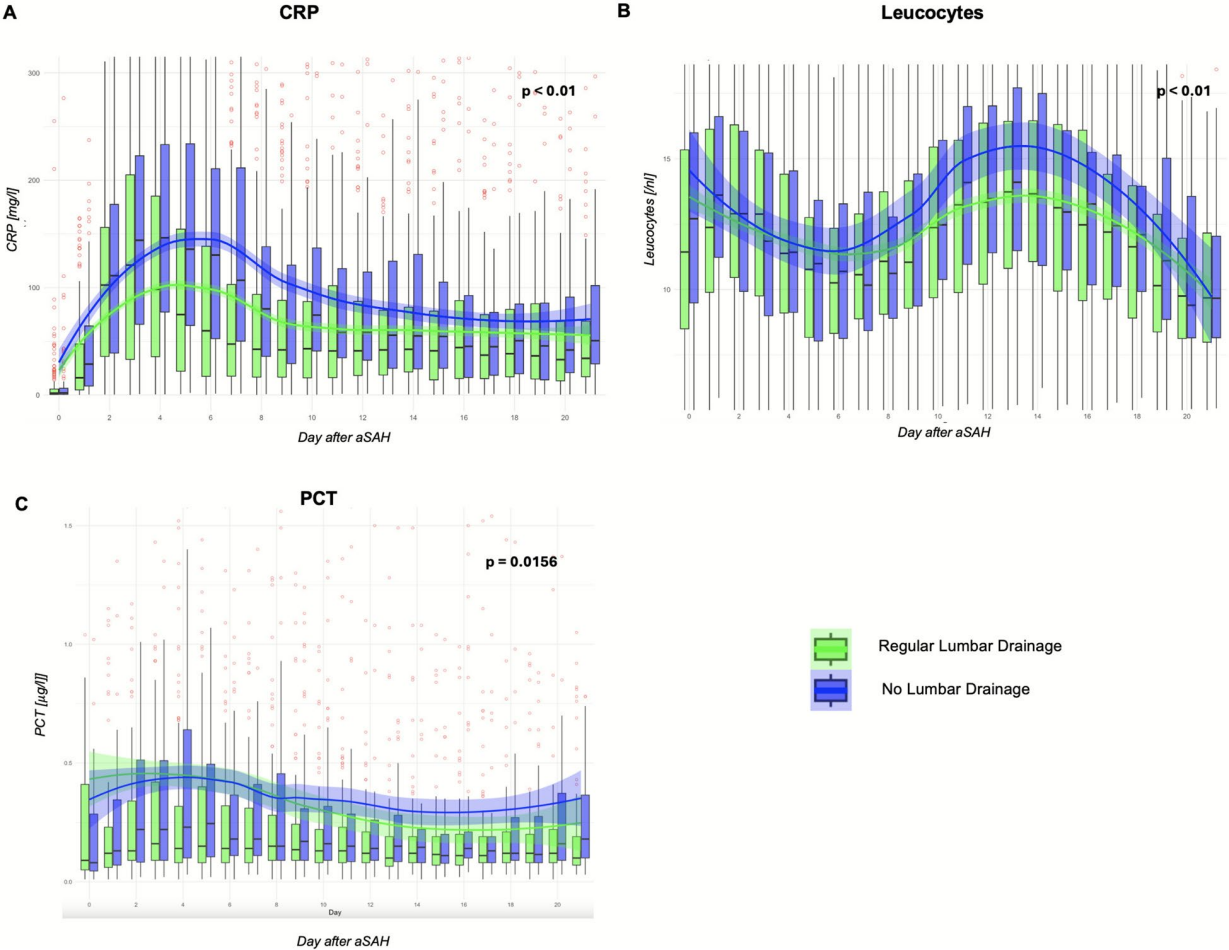
Acute-phase proteins after aneurysmal subarachnoid hemorrhage (aSAH), stratified by patients with or without additional lumbar drainage. **a**, C-reactive protein (CRP). **b**, Leucocytes. **c**, Procalcitonin (PCT). Data are presented as boxplots for the days after aSAH, with outliers highlighted in red. The median trend is shown using a Locally Estimated Scatterplot Smoothing curve, with shaded areas indicating the confidence interval

Patients were stratified based on whether a controlled lumbar drainage was performed or not. The corresponding baseline characteristics are provided in Table 1. No significant differences were observed between the groups regarding the Hunt & Hess grade or age. Figure 5 illustrates the trajectories of leukocyte, CRP, and PCT levels between the groups. All patients showed an initial increase of acute-phase protein levels, with a decline afterward, and these dynamics revealed a significant effect of lumbar drainage. Lower CRP levels were observed when using a lumbar drain (*p* < 0.001). Furthermore, CRP levels peaked earlier and consecutively declined earlier with the use of lumbar drains (*p* < 0.001). Similarly, the analysis of leukocyte dynamics demonstrated a significant association with lumbar drainage, with higher leukocyte levels in the group without drainage (*p* = 0.002). Leukocyte levels also declined significantly over time (*p* = 0.035). In contrast, the analysis of PCT dynamics revealed no significant effect of lumbar drainage (*p* = 0.156). However, PCT levels demonstrated a highly significant decrease over time (*p* < 0.001).

### Infections of the Study Population

To characterize potential infections within the study population, tracheobronchial secretions, blood cultures, and CSF cultures were analyzed. Within the first 14 days following the hemorrhagic event, 10 patients (2.2%) exhibited positive CSF culture results, whereas 279 patients (61%) showed no microbiological growth. In 165 patients (36%), no CSF microbiological data were available, which may be attributable to the absence of CSF sampling or missing data. Among the blood cultures, 3.3% of patients exhibited positive microbial growth, whereas tracheobronchial secretions showed positive culture results in 51% of cases. An overview of the microbiological screening of the study population is presented in Table 2.

**Table 2:** Microbiological screening of the study population during the first 14 days after aneurysmal subarachnoid hemorrhage

	*N* = 454
*CSF, n (%)*	
No CSF sent/available	165 (36)
Negative	279 (61)
Positive	10 (2.2)
*Bacillus circulans*	1 (0.2)
*Candida albicans*	1 (0.2)
*Staphylococcus capitits*	2 (0.4)
*Staphylococcus epidermidis*	4 (0.9)
*Staphylococcus hominis*	1 (0.2)
*Staphylococcus warneri*	1 (0.2)
*Blood culture, n (%)*	
No blood culture sent/available	115 (25)
Negative	324 (71)
Positive	15 (3.3)
*Acinetobacter baumannii*	1 (0.2)
*C. albicans*	2 (0.4)
*Paenibacillus barengoltzii*	1 (0.2)
*Propionibacterium acnes*	1 (0.2)
*Proteus mirabilis* and *Proteus vulgaris*	1 (0.2)
*Serratia marcescens*	1 (0.2)
*Staphylococcus aureus*	2 (0.4)
*Staphylococcus epidermidis*	3 (0.7)
*Stenotrophomonas maltophilia*	1 (0.2)
*Staphylococcus epidermidis* and *Staphylococcus saccharolyticus*	1 (0.2)
*Staphylococcus haemolyticus*	1 (0.2)
*TBS, n (%)*	
No TBS culture sent/available	152 (33)
Negative	72 (16)
Positive	230 (51)

CSF, cerebral spinal fluid, TBS, tracheobronchial secretion

## Discussion

The principal finding of this study is that the majority of blood cells within the CSF system accumulate in the lumbar compartment. The data further demonstrate that the lumbar CSF compartment differs significantly from the ventricular compartment up to the fifth day post bleeding. Therefore, it is tempting to speculate that blood cells in the lumbar compartment may promote inflammatory processes after aSAH. Given compartmental differences, clinicians should carefully consider the origin of CSF samples throughout the clinical management of aSAH to ensure accurate interpretation and optimal therapeutic decision-making.

The course of CSF parameters following aSAH has been described in the literature; however, distinctions between the ventricular and lumbar compartments are often not made. The ranges of these trajectories are comparable to those observed in the present data [14]. Virta et al. [15] retrospectively analyzed single measurements after aSAH from either the ventricular or lumbar compartment and demonstrated large differences between both, showing that measurements are not mutually exchangeable. However, almost no simultaneous CSF comparisons from both compartments were available due to a different clinical approach, and thus there was a potential selection bias of patients for either CSF drainage method. The present study includes data from patients who simultaneously underwent both lumbar and ventricular CSF sampling, providing additional insights into the differences between these compartments.

The CSF space is a self-contained compartment. In the context of aSAH, clinical efforts primarily focus on the cranial compartment, with particular emphasis on washing out the basal cisterns. In contrast, the lumbar compartment previously received less attention. Several studies have hypothesized a role of proinflammatory markers and blood cells in the development of vasospasm and delayed cerebral ischemia [16–19]. The present data suggest that the lumbar compartment may serve as a site of cell accumulation, potentially triggering proinflammatory processes of the innate immune system, such as neutrophil extracellular traps [20]. This finding could also provide a mechanistic explanation for the results of the Earlydrain trial [8], as well as the observed potentially volume-dependent effects of lumbar drainage [9]. Furthermore, the data align with the findings of Bissolo et al., who demonstrated that systemic leukocytosis can be mitigated through intracranial blood clearance achieved via stereotactic catheter ventriculocisternostomy and consecutive irrigation following aSAH [21]. Importantly, EVDs are primarily used for the treatment of acute hydrocephalus and intracranial pressure control, rather than for targeted clearance of the basal cisterns. Alternative approaches, such as cisternostomy or the placement of cisternal drains, have been proposed to enhance cisternal clearance, improve CSF dynamics, and directly remove subarachnoid blood from the basal cisterns [22, 23].

The data also highlight an important consideration for centers that exclusively use an EVD. If a CSF sample is collected after the placement of the EVD, the drain is removed after a few days, and a lumbar puncture is performed later, the elevated cell count in the lumbar sample could be mistakenly interpreted as an infection. Due to the retrospective study design, assessing the patient cohort regarding preexisting infections is challenging. Therefore, we opted for a conservative definition of infection, with a mandatory microorganism detection. The conducted microbiological screening revealed microbial growth in only 2.2% of all patients. We are aware that the actual number of infections is likely to be biased by false positive contamination as well as false negative microbiological testing due to a patient being treated with antibiotics for other sources (most likely pneumonia). Of note, despite extensive research, there is no single laboratory parameter with sufficient yield for the differentiation of device-associated meningitis from sterile inflammation [24].

Regarding serum blood parameters, thrombocytosis is observed after two weeks, which is most likely to be interpreted as a known reactive thrombocytosis. The decrease in hemoglobin levels could be attributed to dilution effects caused by volume therapy administered for blood pressure management and is in line with other studies [25]. The increase in sodium levels during the first week of therapy might be explained by the use of sodium chloride as part of intracranial pressure management or the transient occurrence of diabetes insipidus, a complication observed in aSAH cases [26].

When interpreting the results of this study, several limitations must be acknowledged. Firstly, the analysis concerning treatment modalities is susceptible to selection bias, as it reflects the decisions made by treating physicians. We did not use the presence of infection or similar conditions as an exclusion criterion, as distinguishing central fever from other causes is challenging. However, our data focuses on the initial days after aSAH. Infections usually develop after the first week of drainage use [27]. Although we acknowledge that potential infections introduce potential bias, we do think that infections do not invalidate our findings. The incomplete availability of microbiological CSF data, resulting from the absence of systematic microbiological screening, may limit the interpretation of inflammatory markers. As this is a retrospective study, it is not always clear why certain samples were collected, which limits the ability to standardize the data. In addition, the majority of CSF samples originated from lumbar drains. This may reflect routine clinical practice, in which lumbar drains typically remain in place longer than EVDs and allow easier and safer sampling. This may have introduced a sampling bias toward the lumbar compartment. However, given that lumbar drainage is likely to facilitate the clearance of blood cells, the described accumulation of erythrocytes in the lumbar compartment may actually be underestimated in this study. Patient mobilization represents another potential confounding factor that was not systematically assessed in this retrospective analysis. In our institution, early mobilization and physiotherapeutic exercises are routinely initiated after the aneurysm is secured, if permitted by the patient’s neurological condition. It is conceivable that mobilization may influence the distribution of blood cells within the CSF compartments. This aspect warrants systematic investigation in future prospective studies. We do not have a structured long-term outcome assessment determined at a fixed time point for all patients. We do not see that as serious limitation given the availability of the Earlydrain data; however, this limits the ability to directly correlate early CSF dynamics in the different compartments with long-term neurological outcome of the cohort. Furthermore, correlations between CSF and serum inflammatory markers with the occurrence of vasospasm or delayed cerebral ischemia were not analyzed. This aspect represents an important and intriguing direction for future investigations.

## Conclusions

The primary outcome of our study reveals that blood cells accumulate in the lumbar CSF compartment after aSAH. These cells may promote inflammatory processes. Given compartmental differences, clinicians should carefully consider the origin of CSF samples throughout the clinical management of aSAH to ensure accurate interpretation and optimal therapeutic decision-making.
